# Engineering *Kluyveromyces marxianus* for efficient 2-phenylethanol production: from metabolic design to process innovation

**DOI:** 10.3389/fmicb.2026.1939398

**Published:** 2026-09-17

**Authors:** Yibo Ning, Changtong Lu, Qingfu Wang, Yongzhen Zhao, Zening Wang, Xueying Cao, Haoyang Chen, Tao Wei, Xiaodong Pei, Xiaoxiao Jiang, Feng Zhang, Zhifei Chen, Xu Yang

**Affiliations:** 1 China Tobacco Henan Industrial Co. Ltd., Zhengzhou, China; 2 College of Food and Bioengineering, Zhengzhou University of Light Industry, Zhengzhou, China

**Keywords:** 2-Phenylethanol, CRISPR-Cas, Ehrlich pathway, *Kluyveromyces marxianus*, metabolic engineering

## Abstract

2-Phenylethanol (2-PE) is a valuable aromatic alcohol known for its rose-like scent, widely used in the fragrance, food, and cosmetics industries. The growing demand for “natural” certified products is driving increasing interest in microbial fermentation as a sustainable and promising alternative to chemical synthesis and plant extraction. *Kluyveromyces marxianus* stands out as a promising microbial host for biomanufacturing due to its thermotolerance, rapid growth, versatile substrate use, and ability to catabolize L-phenylalanine (L-Phe) via the Ehrlich pathway. This review synthesizes recent advances in engineering *K. marxianus* for competitive 2-PE production, covering both metabolic design and process innovation. It first examines metabolic engineering strategies to build a robust *K. marxianus* cell factory. These strategies include enhancing central carbon metabolism and precursor biosynthesis, engineering key Ehrlich pathway enzymes to reduce feedback inhibition, optimizing cofactors for redox balance, and improving product tolerance and efflux mechanisms. Looking forward, systems-biology tools—such as multi-omics platforms, genome-scale metabolic models (GSMs), and machine learning—combined with synthetic biology are expected to further rationalize strain design; early applications in *K. marxianus* (e.g., machine-learning-guided 5′ untranslated region optimization) have already been demonstrated, whereas most modeling- and AI-driven strategies for 2-PE production specifically remain at a prospective stage. It also evaluates advances in fermentation process development, including *in situ* product recovery (ISPR), co-fermentation strategies, and sustainable production from renewable, low-cost feedstocks. By integrating recent progress and analyzing challenges in yield optimization, cellular tolerance, and industrial scalability, this review offers a systematic framework for developing efficient, economically viable, and sustainable 2-PE biomanufacturing processes through the integration of molecular and process-level engineering.

## 1 Introduction

2-Phenylethanol (2-PE) is a vital aromatic alcohol known for its lasting, delicate rose scent, making it a highly sought-after flavor compound in the global fragrance industry (Sun et al., 2025). It serves as a crucial component in perfumes, cosmetics, and personal care products, and is extensively used as a flavor additive in food and beverages (Abreu et al., 2023). Recently, the increasing consumer demand for “clean label” and “naturally sourced” products has widened the price gap between natural 2-PE—sourced from plants, animals, or microorganisms through physical or biological processes—and its synthetic version (Yu et al., 2025; Huang et al., 2026). The market value of natural 2-PE can be nearly 300 times higher than that of synthetic products (approximately US$1,000/kg vs. US$3.5/kg), driving advancements in biomanufacturing technologies (Lindquist et al., 2015). Currently, 2-PE is industrially produced through three main methods: chemical synthesis, plant extraction, and microbial fermentation (Wang et al., 2018; Zhan et al., 2023) (Figure 1). Chemical synthesis methods, such as the Friedel–Crafts reaction and the phenyl epoxide pathway, are cost-effective and scalable but are classified as “artificial flavorings,” disqualifying them from “natural” certification (Qian et al., 2019). These processes may also use hazardous reagents and produce undesirable by-products (Wang et al., 2018). Plant extraction, primarily from floral essential oils like rose and jasmine, yields high-quality natural 2-PE but is constrained by the very low recoverable abundance of 2-PE in plant biomass: although 2-PE can account for up to ~60% of rose essential oil by composition (Gu et al., 2020; Dai et al., 2021), the oil itself is present in petals at only trace levels (typically <0.1% of fresh flower weight), such that several tonnes of petals are needed to obtain 1 kg of oil. Together with high land and labor costs and reliance on climate and seasonal variations, this keeps the extracted product costly and impractical for large-scale industrial applications. Faced with these challenges, microbial fermentation has become a sustainable and eco-friendly method for producing naturally certified 2-PE. This approach uses microbial cell factories to transform renewable sugar substrates into 2-PE through intracellular metabolic pathways, mainly the Ehrlich pathway (Zhao et al., 2024). Due to its scalability, sustainability, and alignment with the market demand for natural products, microbial fermentation is increasingly seen as a promising option for future industrial production.

**Figure 1:**
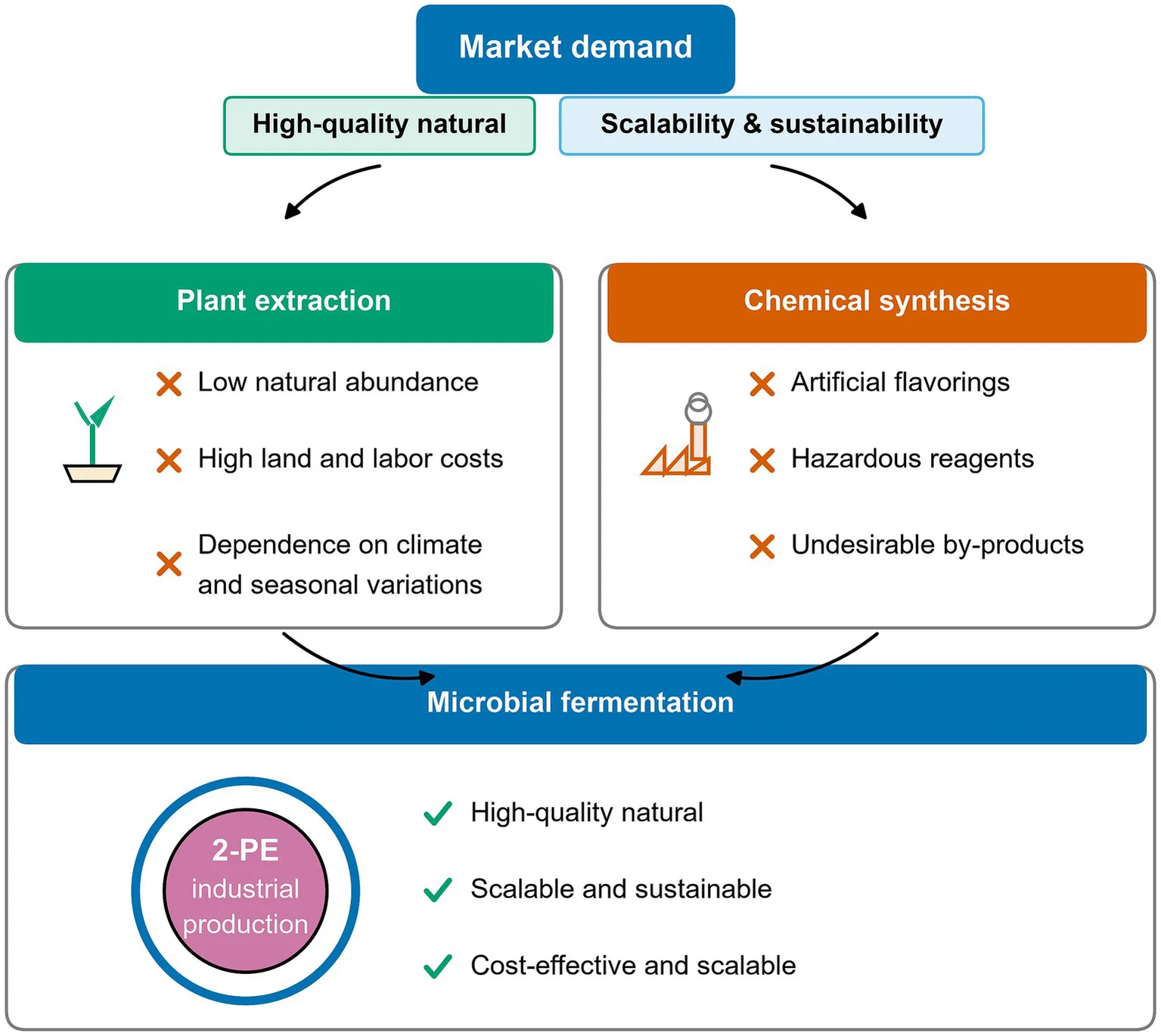
Industrial production of 2-PE approaches: Chemical synthesis, plant extraction, and microbial fermentation.

*Kluyveromyces marxianus* has emerged as a promising microbial host for producing 2-PE and other high-value compounds (Table 1). Compared to the model yeast *Saccharomyces cerevisiae*, *K. marxianus* offers distinct physiological advantages that boost its industrial use (Monteiro et al., 2020; Qiu et al., 2023). It exhibits exceptional thermotolerance, thriving at 40–45 °C and remaining viable above 52 °C (Zhang M. et al., 2025). This trait reduces cooling costs in large-scale fermentation, minimizes contamination risks from mesophilic microorganisms, and aligns with enzyme operating temperatures, enhancing overall process efficiency. Additionally, *K. marxianus* is among the fastest-growing eukaryotes, with a doubling time under 1 h, allowing for shorter fermentation cycles and increased productivity (Wang H. et al., 2025). It can metabolize a wide range of substrates, including glucose, galactose, lactose, inulin, xylose, and hemicellulose-derived disaccharides from lignocellulosic hydrolysates (Patetko et al., 2016; Sharma et al., 2025). This versatility enables the cost-effective use of renewable resources like whey, chicory root extract, and agricultural-waste hydrolysates, reducing raw-material costs and supporting circular-economy strategies. Moreover, *K. marxianus* is generally recognized as safe (GRAS) and has a well-characterized genetic background (Zou et al., 2025). It naturally possesses a phenylalanine-degradation pathway, known as the Ehrlich pathway, which provides a metabolic foundation for 2-PE biosynthesis (Rajkumar and Morrissey, 2020).

**Table 1:** Advances in the synthesis of 2-phenylethanol in some microorganisms.

Host	Substrate	Max titer	Productivity	Fermentation modes	Reference
*Saccharomyces bayanus* L1	Glucose L-Phenylalanine	4.2 g/L	0.058 g/L/h	Batch liquid fermentation	Zhao et al. (2024)
		6.5 g/L	0.108 g/L/h	Fed-batch fermentation	
*Pichia kudriavzevii* YF1702	Glucose L-Phenylalanine	5.09 g/L	0.09 g/L/h	Batch liquid fermentation	Fan et al. (2020)
*Zygosaccharomyces rouxii* M2013310	Glucose L-Phenylalanine	3.58 g/L	0.05 g/L/h	Batch liquid fermentation	Dai et al. (2020)
*Acinetobacter soli* ANG344B	Glucose L-Phenylalanine	2.35 g/L	0.10 g/L/h	Batch liquid fermentation	Bernardino et al. (2024a)
Engineered *Escherichia coli* BL21	Glucose	320 mg/L	13.3 mg/L/h	Batch liquid fermentation	Liu et al. (2018)
Engineered *Bacillus licheniformis* DE4	Glucose L-Phenylalanine	5.16 g/L	0.12 g/L/h	Fed-batch fermentation	Zhan et al. (2020)
Engineered *Bacillus licheniformis* PE23	Glucose L-Phenylalanine	6.24 g/L	0.13 g/L/h	Fed-batch fermentation	Zhan et al. (2022)
Engineered *Bacillus licheniformis* PE-4	Glucose L-Phenylalanine	6.95 g/L	0.14 g/L/h	Batch liquid fermentation	Rao et al. (2023)
Engineered *Corynebacterium glutamicum* CGPE15	Corn stover hydrolysate	3.28 g/L	0.07 g/L/h	Batch liquid fermentation	Zhu et al. (2023)
Engineered *Saccharomyces cerevisiae* YS58	Glucose L-Phenylalanine	6.30 g/L	0.05 g/L/h	Fed-batch fermentation	Wang et al. (2018)
Engineered *Saccharomyces cerevisiae* FY202001	Glucose L-Phenylalanine	4.93 g/L	0.103 g/L/h	Batch liquid fermentation	Xia et al. (2022b)
Wild-Type *Saccharomyces cerevisiae* D-22	Glucose L-Phenylalanine	6.41 g/L	0.267 g/L/h	Fed-batch fermentation *In situ* product recovery (ISPR)	Zhang C. S. et al. (2025)
Engineered *Saccharomyces cerevisiae* IMX2179	Glucose L-Phenylalanine	1.59 g/L	0.04 g/L/h	Batch liquid fermentation	Hassing et al. (2019)
*Saccharomyces cerevisiae* JM2014	Glucose L-Phenylalanine	9.79 g/L	135.97 mg/L/h	Batch liquid fermentation In situ product recovery	Chreptowicz and Mierzejewska (2018)
Engineered *Kluyveromyces marxianus* KmTY4	Glucose	1.30 g/L	0.018 g/L/h	Batch liquid fermentation	Kim et al. (2014)
*Metschnikowia pulcherrima* NCYC 373	Glucose L-Phenylalanine	14.0 g/L	0.029 g/L/h	Continuous fermentation Solid phase extraction	Chantasuban et al. (2018)
*Pichia kudriavzevii* CECT 13184	Fermented sugarcane bagasse L-Phenylalanine	27.2 mg/g	0.72 mg/g/h	Sequential-batch SSF	Martínez-Avila et al. (2020)
Engineered *Escherichia coli* DH5α	Glucose L-Phenylalanine	2.15 g/L	0.067 g/L/h	Fed-batch fermentation	Wang et al. (2022)
Engineered *Escherichia coli CFT3*	Glucose L-Phenylalanine	2.5 g/L	0.035 g/L/h	Batch liquid fermentation	Noda et al. (2024)
*Kluyveromyces marxianus* ATCC 10022	Sugarcane bagasse L-Phenylalanine	18.4 mg/g	22.65 mg/L/h	Sequential-batch SSF	Martínez et al. (2018)
*Pichia kudriavzevii* CECT 13184	Red apple pomace L-Phenylalanine	25.2 mg/g	0.36 mg/g/h	SSF	Martínez-Avila et al. (2021)
Engineered *Saccharomyces cerevisiae IMX2179*	Glucose	16.6 g/kg_SW_	0.16 g/kg_SW_/h	Fed-batch fermentation Continuous extractive	Brewster et al. (2025)
Engineered *Rhodotorula toruloides* NP11	Glucose	1.06 g/L	8.0 mg/L/h	Batch liquid fermentation	Zheng et al. (2024)
Engineered *Candida glycerinogenes* WL2002-5	Glucose L-Phenylalanine	5.0 g/L	0.21 g/L/h	Batch liquid fermentation In situ product recovery	Wang et al. (2020)
*Kluyveromyces marxianus* WUT240	Whey Permeate	1.12 g/L	57.5 mg/L/h	Continuous system	Drezek et al. (2021)
Engineered *Escherichia coli* strain 6	Glucose	7.4 g/L	0.744 g/L/h	Batch liquid fermentation	Xiong et al. (2025)
*Kluyveromyces marxianus* NRRL Y-1109	Bark white pine wood waste	1.09 g/L	0.011 g/L/h	Batch liquid fermentation	Pachapur et al. (2024)
Co-culture *Kluyveromyces marxianus* NRRL Y-1109 and *Debaryomyces hansenii* NRRL Y-1448	Cheese whey	2.0 g/L	0.04 g/L/h	Batch liquid fermentation	Castillo et al. (2023)
Co-culture *Kluyveromyces marxianus* MUCL 53775 and *Meyerozyma guilliermondii* MUCL 28072	Glucose L-Phenylalanine	1,168 mg/L	24.3 mg/L/h	*In Situ* Product Recovery (absorptive polymer Hytrel3548)	Lukito et al. (2023)

Values compiled from studies that differ in host strain, substrate (glucose alone vs. L-phenylalanine-supplemented), cultivation mode, and use of in situ product recovery (ISPR); titers and productivities are therefore indicative benchmarks rather than directly comparable performance metrics.

Despite *K. marxianus*’s significant potential, native strains still do not produce 2-PE at levels sufficient for industrial demand (Rogalska et al., 2025). To overcome these limitations, clearer engineering strategies are essential for guiding researchers toward more effective solutions. Substantial progress requires a comprehensive, multi-scale systems engineering approach that integrates both intracellular and extracellular optimization. Recent research has evolved from simple gene overexpression to multidimensional strategies combining metabolic engineering, systems biology, and innovative fermentation technologies. This review provides a systematic and forward-looking perspective on engineering *K. marxianus* throughout the technological chain—from metabolic design to process innovation—to enable efficient, cost-effective, and sustainable 2-PE production. Initially, the article examines intracellular metabolic engineering strategies, including pathway optimization, improved precursor supply, and enhanced stress tolerance. It then explores how systems biology tools enable rational design, followed by a discussion of key advances in fermentation processes, such as novel operating modes and the use of low-cost substrates. Finally, it analyzes significant current challenges and outlines future research directions. This framework offers researchers and engineers a clear technical roadmap to accelerate the transition of *K. marxianus*-based 2-PE biomanufacturing from laboratory research to industrial application.

Several previous reviews have summarized biotechnological 2-PE production across multiple hosts (Qian et al., 2019; Bernardino et al., 2024b) or the general biotechnological potential of *K. marxianus* (Fonseca et al., 2008; Qiu et al., 2023; Wang H. et al., 2025). However, no prior review has systematically connected *K. marxianus*-specific metabolic design with process-level innovation specifically for 2-PE. The present review fills this gap by (i) integrating strain engineering—precursor supply, Ehrlich-pathway optimization, cofactor balance, and tolerance engineering—with fermentation and downstream strategies (ISPR, co-cultivation, low-cost feedstocks) within a single coherent framework; (ii) providing quantitative benchmarking of *K. marxianus* against alternative production hosts; and (iii) framing remaining challenges and research directions according to their technical maturity, from experimentally demonstrated strategies to prospective opportunities.

## 2 Metabolic engineering: rewiring the intracellular factory

Efficient microbial cell factories rely on the intentional design and remodeling of cellular metabolic networks (Zhang X. J. et al., 2025). Metabolic engineering serves as the core framework for this process. By specifically modifying key biochemical reactions, it systematically refines metabolic pathways, transforming host cells into effective catalysts for synthesizing desired products. In producing 2-PE with *K. marxianus* as the chassis organism, metabolic engineering efforts primarily concentrate on interconnected dimensions, as illustrated in Figures 2A–C and discussed below (precursor supply, Ehrlich-pathway engineering, cofactor balance, and tolerance/efflux).

**Figure 2:**
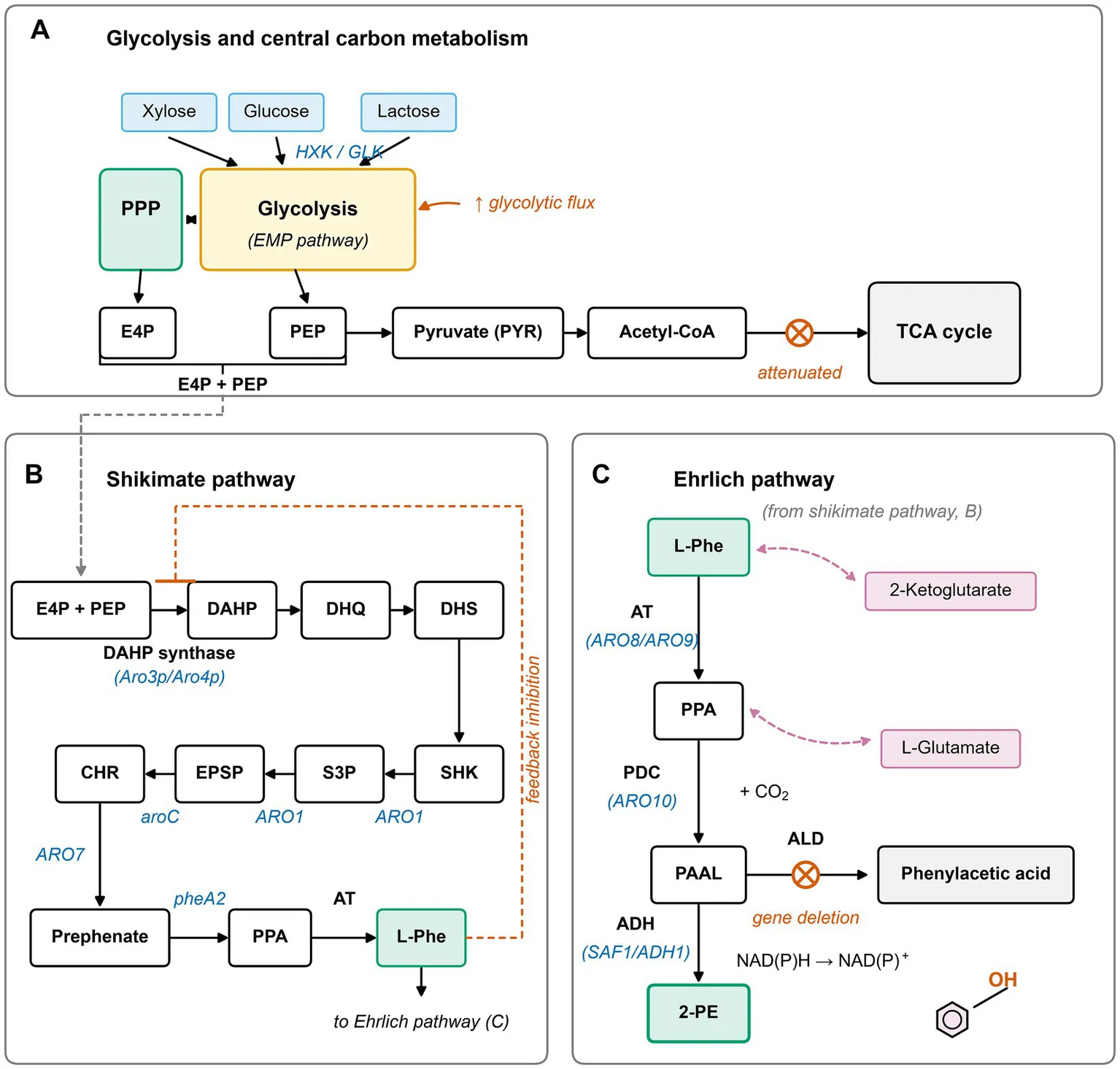
Biosynthesis of fragrance 2-PE by *K. marxianus*. **(A)** Glycolysis and central carbon metabolism, showing carbon flux from glucose through the EMP pathway to pyruvate and acetyl-CoA; **(B)** The shikimate pathway, depicting the conversion of E4P and PEP through seven enzymatic steps to chorismate and subsequently to L-phenylalanine; **(C)** The Ehrlich pathway, illustrating the three-step conversion of L-Phe to 2-PE via phenylpyruvate (transamination), phenylacetaldehyde (decarboxylation), and 2-PE (reduction). HXK, hexokinase; GLK, glucokinase; TCA, tricarboxylic acid; PPP, pentose phosphate pathway; PEP, phosphoenolpyruvate; PYR, pyruvate; E4P, erythrose-4-phosphate; DAHP, 3-deoxy-D-arabinoheptulosonate; DHQ, 3-dehydroquinate; DHS, 3-dehydroshikimate; SHK, shikimate; S3P, shikimate-3-phosphate; EPSP, 5-enolpyruvyshikimate-3-phosphate; CHR, chorismate; PPA, phenylpyruvate; PAAL, phenylacetaldehyde; 2-PE, 2-phenylethanol; L-Phe, L-phenylalanine; AT, transaminase; PDC, phenylpyruvate decarboxylase; ALD, aldehyde dehydrogenase; ADH, alcohol dehydrogenase.

### 2.1 Enhancing central carbon metabolism and precursor supply

The efficient synthesis of 2-PE relies on maintaining adequate and properly directed carbon flux within cells (Wu et al., 2023). Metabolic engineering aims to enhance central carbon metabolism to provide sufficient precursors and energy for 2-PE biosynthesis (Figure 2A). A common strategy to increase key intermediates like acetyl-CoA and pyruvate is to enhance glycolytic flux. Research indicates that overexpressing regulatory factors or rate-limiting enzymes in the glycolytic pathway of *K. marxianus* significantly accelerates carbon-source utilization. For instance, strains engineered to overexpress hexokinase (HXK) and glucokinase (GLK) demonstrate faster glucose consumption and improved growth during high-temperature fermentation, thus supplying a more robust carbon source for downstream synthetic pathways (Zhang et al., 2017; Wang et al., 2023). Reducing glucose catabolic inhibition further facilitates the efficient use of mixed sugars, such as glucose and lactose, which is crucial for converting low-cost substrates like whey (Drezek et al., 2023). Regulating the acetyl-CoA pool is also critical, as acetyl-CoA is a central metabolite in energy metabolism. Strategies demonstrated in *S. cerevisiae*—overexpressing the pyruvate dehydrogenase complex or introducing engineered acetyl-CoA synthase to increase cytosolic acetyl-CoA (Chen et al., 2013), and attenuating citrate synthase activity to limit acetyl-CoA diversion into the tricarboxylic acid cycle (Meadows et al., 2016)—can, in principle, be transferred to *K. marxianus*. To date, however, these interventions have not been experimentally demonstrated for 2-PE production in *K. marxianus*, and they are therefore proposed here as rational, transferable strategies rather than established approaches in this host (Nielsen and Keasling, 2016).

Optimizing the biosynthesis of aromatic amino acid precursors provides a direct strategy. L-phenylalanine (L-Phe), the immediate precursor of 2-PE, originates from the shikimate pathway (Figure 2B) (Godoy et al., 2024). Enhancing its initial step, particularly by overexpressing feedback-insensitive 3-deoxy-D-arabino-heptulosonate-7-phosphate (DAHP) synthase encoded by Aro3p and Aro4p, has significantly increased the accumulation of shared aromatic amino acid precursors (de Lima et al., 2021). Strengthening the non-oxidative pentose phosphate pathway to boost erythrose-4-phosphate (E4P) availability and facilitate its efficient condensation with phosphoenolpyruvate (PEP) is crucial for alleviating upstream bottlenecks (Rajkumar and Morrissey, 2020). Recent systematic metabolic engineering studies have employed modular strategies to optimize enzyme expression across glycolysis, the pentose phosphate pathway, and the shikimate pathway (González-Lozano et al., 2025). This coordinated approach balances metabolic fluxes and prevents growth inhibition caused by intermediate accumulation.

*K. marxianus* has unique metabolic traits that set it apart from *S. cerevisiae* and other yeasts. It features a distinct carbon catabolite repression (CCR) mechanism, unlike the well-known system in *S. cerevisiae*. While *S. cerevisiae* strongly represses alternative sugar utilization genes in the presence of glucose, *K. marxianus* shows a partially relaxed CCR, allowing it to simultaneously utilize glucose and xylose (Kim et al., 2019). This trait is particularly beneficial for producing 2-PE from lignocellulosic hydrolysates. Moreover, *K. marxianus*’s thermotolerance is linked to its membrane lipid composition, which maintains fluidity and integrity at higher temperatures (40–45 °C). Modifying membrane ergosterol content and fatty acid profiles has been shown to enhance both thermotolerance and 2-PE tolerance, as both stresses affect membrane integrity (Balbino et al., 2021). Additionally, *K. marxianus* has unique stress response pathways, including strong oxidative stress resistance mediated by thioredoxin systems. Overexpression of its thioredoxin system (thioredoxin and thioredoxin reductase) has been experimentally shown to enhance tolerance to multiple lignocellulose-derived inhibitors (Gao et al., 2017); whether this system can likewise mitigate 2-PE toxicity is a plausible but not yet demonstrated extension.

### 2.2 Engineered Ehrlich pathway and feedback inhibition relief

The Ehrlich pathway is the main route by which yeast converts L-Phe into 2-PE, involving three enzymatic reactions: transamination, decarboxylation, and reduction (Figure 2C). Precise engineering of this pathway is vital for enhancing 2-PE yield and productivity. Overexpressing and screening key enzymes are the most direct and effective strategies (Liu et al., 2018). Initial efforts focused on boosting the activity of aromatic aminotransferases (AT), which catalyze the first transamination step. Studies indicate that AT in *K. marxianus* is crucial under conditions like nitrogen limitation; its overexpression significantly increases 2-PE production (Li et al., 2021). Additionally, in *S. cerevisiae*, upregulation of Ehrlich-pathway decarboxylase and alcohol-dehydrogenase genes directs metabolic flux toward 2-PE formation and reduces accumulation of the toxic intermediate phenylacetaldehyde (Yin et al., 2015; Wang et al., 2017; Bernardino et al., 2024b). In *K. marxianus*, functional characterization of the pyruvate decarboxylase genes KmPDC1 and KmPDC5 supports their involvement in phenylpyruvate decarboxylation (Choo et al., 2018); however, systematic co-overexpression of PDC and ADH for 2-PE production has not yet been reported in this host and is therefore proposed as a rational strategy. Blocking competing pathways is also essential for maximizing carbon flux toward the target product (Saha et al., 2024). Phenylacetaldehyde can be oxidized by aldehyde dehydrogenase to phenylacetic acid, releasing carbon (Gu et al., 2020; Yu et al., 2024). Deleting or inhibiting key aldehyde dehydrogenase (ALD) genes effectively suppresses phenylacetic acid formation, redirecting more precursors toward 2-PE synthesis (Holyavkin et al., 2023; Heider and Hege, 2025).

Overcoming intrinsic regulatory constraints requires relieving L-Phe feedback inhibition. In microbial cells, L-Phe allosterically inhibits DAHP synthase, the first enzyme in its biosynthetic pathway (Liu et al., 2023) (Figures 2B,C). Introducing feedback-insensitive DAHP synthase mutants has been a successful strategy. In *K. marxianus*, these mutants increased intracellular L-Phe accumulation, enriching the substrate supply for the Ehrlich pathway. This enabled high 2-PE yields even with low supplemental L-Phe (Kim et al., 2014).

### 2.3 Cofactor engineering and redox balance

The biosynthesis of 2-PE is intricately tied to the intracellular redox balance. In the Ehrlich pathway’s final step, converting phenylacetaldehyde to 2-PE requires the reduced cofactor NAD(P)H, often making the supply of reducing power a rate-limiting factor (Figure 2C). One solution is regulating coenzyme preference. If key reductases, like phenylacetaldehyde reductase, primarily depend on NADPH, maintaining adequate NADPH is crucial. This can be achieved by overexpressing NADPH-generating enzymes in the pentose phosphate pathway, such as glucose-6-phosphate dehydrogenase (Alarcon et al., 2012). Alternatively, engineering these reductases to shift their cofactor preference to utilize the more abundant NADH pool is another strategy. A systematic approach involves establishing efficient cofactor-recycling mechanisms. Introducing an exogenous regeneration system coupled with the product synthesis pathway can sustain redox homeostasis (Sun et al., 2023). For instance, co-expressing formate dehydrogenase while engineering phenylacetaldehyde reductase to switch its cofactor from NADPH to NADH could theoretically create a continuous cycle of reducing-power supply using formate as the electron donor, thereby enhancing 2-PE production efficiency (Lynch et al., 2026).

### 2.4 Enhancing product tolerance and efflux

2-PE is highly toxic to microbial cells, even at low concentrations, as it disrupts membrane integrity and inhibits growth and metabolism. This toxicity is a significant bottleneck that limits final fermentation titers (Zhan et al., 2023; Brewster et al., 2025). Therefore, enhancing host tolerance is not just an optimization step but a fundamental requirement for industrial-scale production.

Modifying cell membrane composition and fluidity is crucial for enhancing tolerance. Membrane integrity serves as the primary defense against hydrophobic organic compounds. In *K. marxianus*, tolerance engineering is beginning to move from general yeast paradigms toward host-specific targets. 2-PE-adapted cells of *K. marxianus* CCT 7735 were shown to remodel their envelope by increasing ergosterol content, shifting fatty-acid composition toward higher unsaturation, and producing exopolysaccharides, which implicates ergosterol-biosynthesis (*ERG*-pathway) genes and fatty-acid desaturases as concrete engineering targets (Balbino et al., 2021). Consistent with this, deliberate alteration of sterol composition through engineering of the ergosterol pathway has been demonstrated to enhance multiple-stress tolerance in *K. marxianus* in a xylitol-production context (Ren et al., 2024), and overexpression of the thioredoxin system (KmTRX1/KmTRR1) improved tolerance to furfural, HMF, and phenolic lignocellulose-derived inhibitors (Gao et al., 2017), although a corresponding demonstration for 2-PE stress remains to be established. In *S. cerevisiae*, combined overexpression of ergosterol-pathway genes such as *ERG1*, *ERG3*, and *ERG11* increases membrane sterol content (Veen et al., 2003; Xie et al., 2026), and a crucial *INO2* allele enhances ethanol resistance in an industrial background (Albillos-Arenal et al., 2025); these paradigms represent promising, transferable targets for *K. marxianus*. Adjusting the phosphocholine-to-phosphoethanolamine ratio or incorporating longer-chain fatty acids has also strengthened yeast tolerance to organic solvents (Yuan et al., 2024).

Engineering active efflux systems offers a direct protective mechanism. Microorganisms naturally have diverse transmembrane transporters that expel toxic substances. In *S. cerevisiae*, gain-of-function mutations in the PDR network that upregulate ABC transporters (e.g., Pdr5p) have been experimentally linked to 2-PE tolerance and export (Xia et al., 2022a; Holyavkin et al., 2023). Homologous MFS- and ABC-family transporters of *K. marxianus* represent promising but not yet validated targets for enhancing 2-PE efflux; their engineering could reduce intracellular toxicity in real time and potentially shift reaction equilibria to sustain metabolic flux.

Adaptive laboratory evolution is a powerful method for uncovering tolerance mechanisms (Dragosits and Mattanovich, 2013; Zhang Y. F. et al., 2025; Declerck et al., 2026). By continuously cultivating wild-type or engineered strains under increasing 2-PE stress, researchers selected mutants with improved tolerance (Yin et al., 2015; Noda et al., 2024; Zhang C. S. et al., 2025). Whole-genome sequencing and reverse engineering then revealed novel genes related to vesicular transport, stress responses, or transcriptional regulation (Zhu et al., 2021; Xia et al., 2022a). These findings provide new targets for rational metabolic engineering.

## 3 Systems biology and emerging tools: towards rational design

Classical metabolic engineering struggles to modify complex traits, prompting a shift in microbial cell factory development from empirical trial-and-error to rational design based on deeper biological understanding. This transition is driven by rapid advancements in systems and synthetic biology. These fields allow researchers to uncover physiological mechanisms with exceptional detail and execute genetic modifications with enhanced precision and efficiency (Malci et al., 2022).

### 3.1 Mechanistic elucidation driven by omics technologies

Systems biology combines data from genomes, transcriptomes, proteomes, metabolomes, and fluxomes to comprehensively understand cellular physiology. In research on engineered *K. marxianus* for 2-PE production, multi-omics analyses are crucial for revealing high-yield mechanisms and identifying new engineering targets (Figure 3).

**Figure 3:**
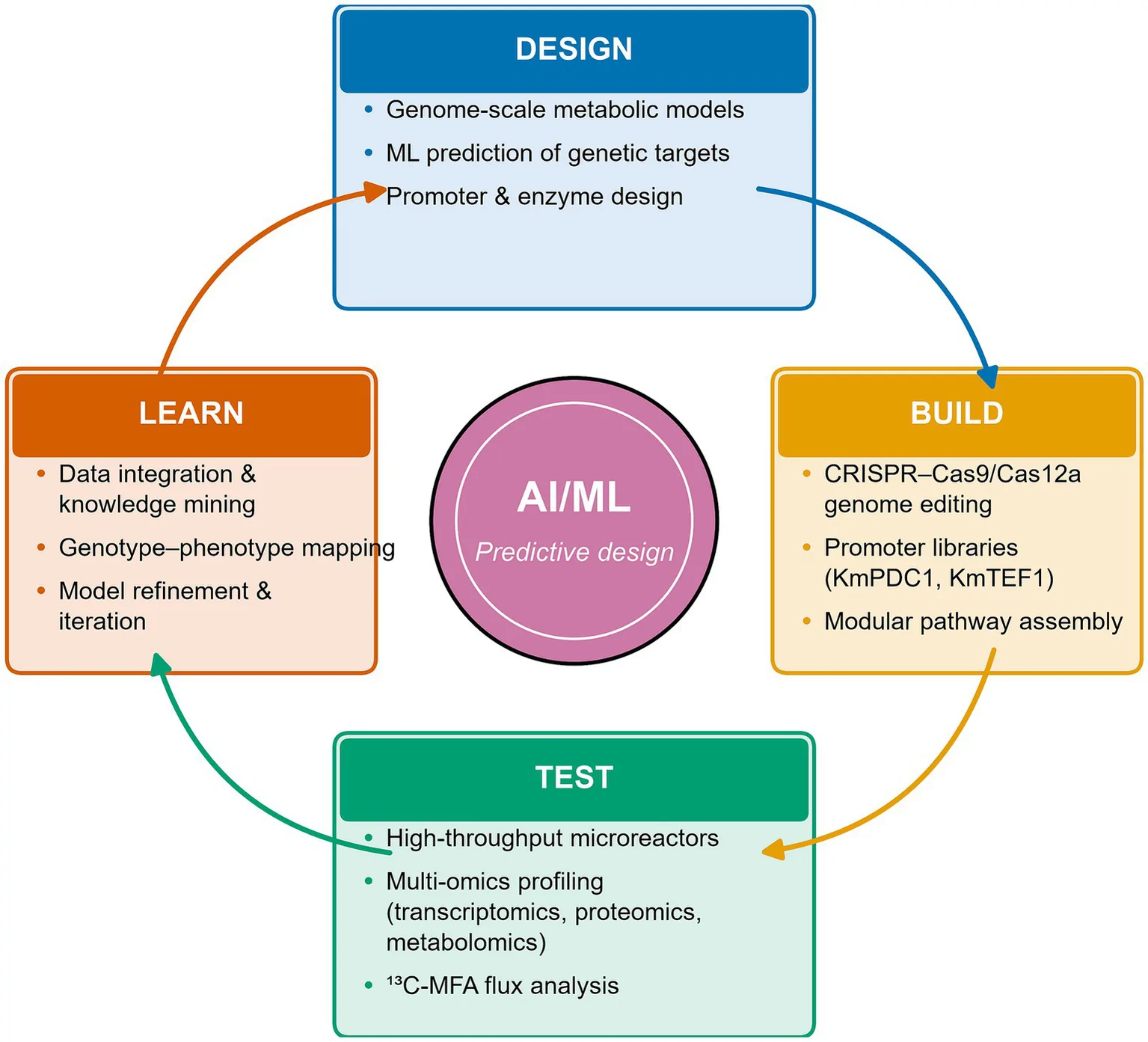
Combining systems metabolic engineering with artificial intelligence (AI)/machine learning (ML) through an iterative design–build–test–learn (DBTL) cycle centered on AI/ML-driven predictive design. The learn phase integrates multi-omics datasets (genomics, transcriptomics, proteomics, metabolomics, and fluxomics) and mines prior engineering knowledge for genotype–phenotype mapping and iterative model refinement; the design phase uses genome-scale metabolic models, machine-learning prediction of genetic intervention targets, and promoter and enzyme design; the build phase employs CRISPR-Cas9/Cas12a genome editing, promoter libraries (e.g., KmPDC1, KmTEF1), and modular pathway assembly; and the test phase relies on high-throughput microreactor cultivation, multi-omics profiling, and 13C-metabolic flux analysis, whose outputs feed back into the learn phase to close the cycle.

Transcriptomic and proteomic approaches are widely used to identify global changes in gene expression and protein synthesis under specific high-production conditions, like nitrogen limitation or product stress (Christopher et al., 2022). A recent time-series transcriptomic study on the *K. marxianus* ITD0090 strain under nitrogen limitation showed that nitrogen signaling coordinates carbon metabolism reprogramming, enhances amino acid biosynthesis, and activates secondary metabolic pathways (González-Lozano et al., 2025). This study reaffirmed the central role of ARO9 and identified synergistically upregulated genes involved in acetyl-CoA supply and esterification, providing a roadmap for multi-target optimization. Comparative proteomics can reveal differences in protein expression between wild-type and high-yield mutant strains, such as those developed through adaptive evolution, identifying key protein modules linked to product tolerance and increased metabolic flux.

Metabolomics and flux analyses offer direct insights into metabolite dynamics and metabolic flux distribution (Jarboe et al., 2010). Quantifying intermediate metabolites in the shikimate and Ehrlich pathways, along with related bypass routes, identifies rate-limiting steps and metabolic branch points precisely (Guo et al., 2019; Xu et al., 2024). For example, metabolomic data may reveal that high-yield strains accumulate significantly less phenylacetaldehyde than low-yield strains, with a concurrent decline in competing metabolites like phenylacetic acid (Yu et al., 2023). These observations indirectly support the expected effects of increased reductase activity and reduced bypass flux. When combined with ^13^C-labeled flux analysis (^13^C-MFA), this method quantitatively maps shifts in carbon allocation within central metabolic networks. It allows for accurate assessment of the impact of metabolic engineering strategies, such as targeted gene overexpression or pathway knockout, on global flux distribution, thereby guiding more refined pathway optimization (Gupta et al., 2017; Steichen et al., 2024).

### 3.2 Advances in synthetic biology tools

Rational design relies on the precise manipulation of genetic material. Recently, significant advancements in synthetic biology tools for the non-traditional yeast *K. marxianus* have greatly enhanced its potential as an engineered cell factory.

Efficient genome-editing technologies are central to recent advances, with CRISPR–Cas systems leading the way. Both CRISPR-Cas9 and CRISPR-Cas12a have been successfully adapted for use in *K. marxianus* (Li et al., 2021; Zha et al., 2026). These systems enable high-efficiency gene knockout, knock-in, and point mutation through guide RNAs and repair templates targeting specific loci. Unlike traditional gene targeting that relies on native homologous recombination—which is typically inefficient in *K. marxianus* because non-homologous end joining dominates double-strand-break repair (often at or below ~1% across common laboratory backgrounds)(Rajkumar and Morrissey, 2020; Malci et al., 2022)—optimized CRISPR-Cas9 and Cas12a systems developed for *K. marxianus* routinely achieve editing efficiencies above 80% and allow simultaneous modification of multiple genes (Li et al., 2021; Zha et al., 2026). We note that these efficiencies depend on the strain background, target locus, donor-template design, and Cas-expression conditions, so the comparison should be read as indicative rather than absolute. This capability significantly accelerates metabolic pathway construction and optimization. Additionally, the development of CRISPR interference and activation systems offers reversible, multiplex control of gene expression without altering genomic DNA, providing valuable tools for dynamic regulatory studies.

Regulatory elements play a crucial role in precise metabolic control. A significant advancement has been the development of promoter libraries for *K. marxianus*, which include constitutive, inducible, and gradient-strength promoters (Malci et al., 2022; Yuzbashev et al., 2023). Engineered promoters, derived from strong endogenous elements like KmPDC1 and KmTEF1, along with heterologous systems regulated by tetracycline or galactose, allow for precise modulation of pathway gene expression at various phases and intensities (Choo et al., 2018; Wang H. et al., 2025; Zhang M. et al., 2025). Additionally, engineered 5′ untranslated regions (5′ UTRs) and translation-initiation elements, terminators, and protein-degradation tags form a multilayered, predictable toolkit for regulating gene expression, enabling fine control of metabolic flux; machine-learning-assisted optimization of 5′ UTRs has been demonstrated in *K. marxianus* (Zeng et al., 2024).

### 3.3 Systems metabolic engineering and machine learning

Systems biology generates large datasets, and synthetic biology tools allow for advanced genetic modifications. The primary goal of merging systems metabolic engineering with artificial intelligence (AI) and machine learning (ML) is to achieve predictive design (Figure 3).

Genome-scale metabolic models (GSMs) serve as a foundational framework for computational simulations and predictions (Gong et al., 2024). A high-quality, experimentally validated GSM for *K. marxianus* effectively simulates cellular metabolic states across various genetic and environmental conditions (Marcisauskas et al., 2019). Using constraint-based modeling, flux balance analysis, and depletion studies, GSMs systematically identify gene targets for knockout, overexpression, or attenuation to boost 2-PE production. This method prioritizes promising engineering strategies for experimental validation, minimizing the need for trial and error.

The integration of machine learning (ML) and artificial intelligence (AI) significantly advances rational design. By creating a relational database that consolidates multi-omics data, strain phenotypes, genetic manipulation histories, and fermentation parameters, ML algorithms like random forests and neural networks can discern complex nonlinear relationships within these datasets (Zeng et al., 2024; Zeng et al., 2025). This data-driven framework enables the identification of previously unrecognized targets linked to productivity or tolerance, predicts optimal genetic combinations by forecasting synergistic effects and avoiding negative trade-offs among numerous modification possibilities, and optimizes fermentation processes by correlating strain genotypes with cultivation conditions, thus recommending customized protocols for engineered strains. Emerging studies have applied ML to genomic data from adaptively evolved strains, swiftly identifying single-nucleotide polymorphisms (SNPs) associated with 2-PE tolerance and integrating these insights into rational genome engineering (Guo et al., 2026).

Machine learning (ML) applications in related yeast systems highlight the potential for engineering *K. marxianus*. In *S. cerevisiae*, ML-guided promoter design has enabled precise control of gene expression, optimizing flux balance in aromatic amino acid pathways (Tang et al., 2020). In *Yarrowia lipolytica*, ML models trained on multi-omics data from adaptively evolved strains have predicted genotype–phenotype relationships for 2-PE tolerance, identifying novel mutations in membrane transport genes (Sahu et al., 2021). Specifically for *K. marxianus*, ML-assisted optimization of 5′ untranslated regions with poly-adenine tracts has improved heterologous gene expression, offering a practical tool for pathway engineering (Zeng et al., 2024). These examples demonstrate that ML can significantly accelerate *K. marxianus* strain engineering when adequate training data are available.

## 4 Process innovation: from lab to scale

Superior engineered strains form the foundation for efficient biomanufacturing. However, transforming their potential into economically viable production demands innovative fermentation strategies and careful process design. The primary goals of these innovations are to maximize spatiotemporal product yield, lower production costs, and enhance overall system sustainability. In the context of *K. marxianus*–based 2-PE production, process innovation emphasizes creating high-efficiency fermentation schemes, utilizing low-cost feedstocks, and advancing systematic process integration and intensification.

### 4.1 High-efficiency fermentation modes

Traditional batch fermentation faces challenges like substrate inhibition, product inhibition, and low final product concentrations (Brewster et al., 2025). Advanced fermentation strategies address these problems by enhancing process control (Figure 4).

**Figure 4:**
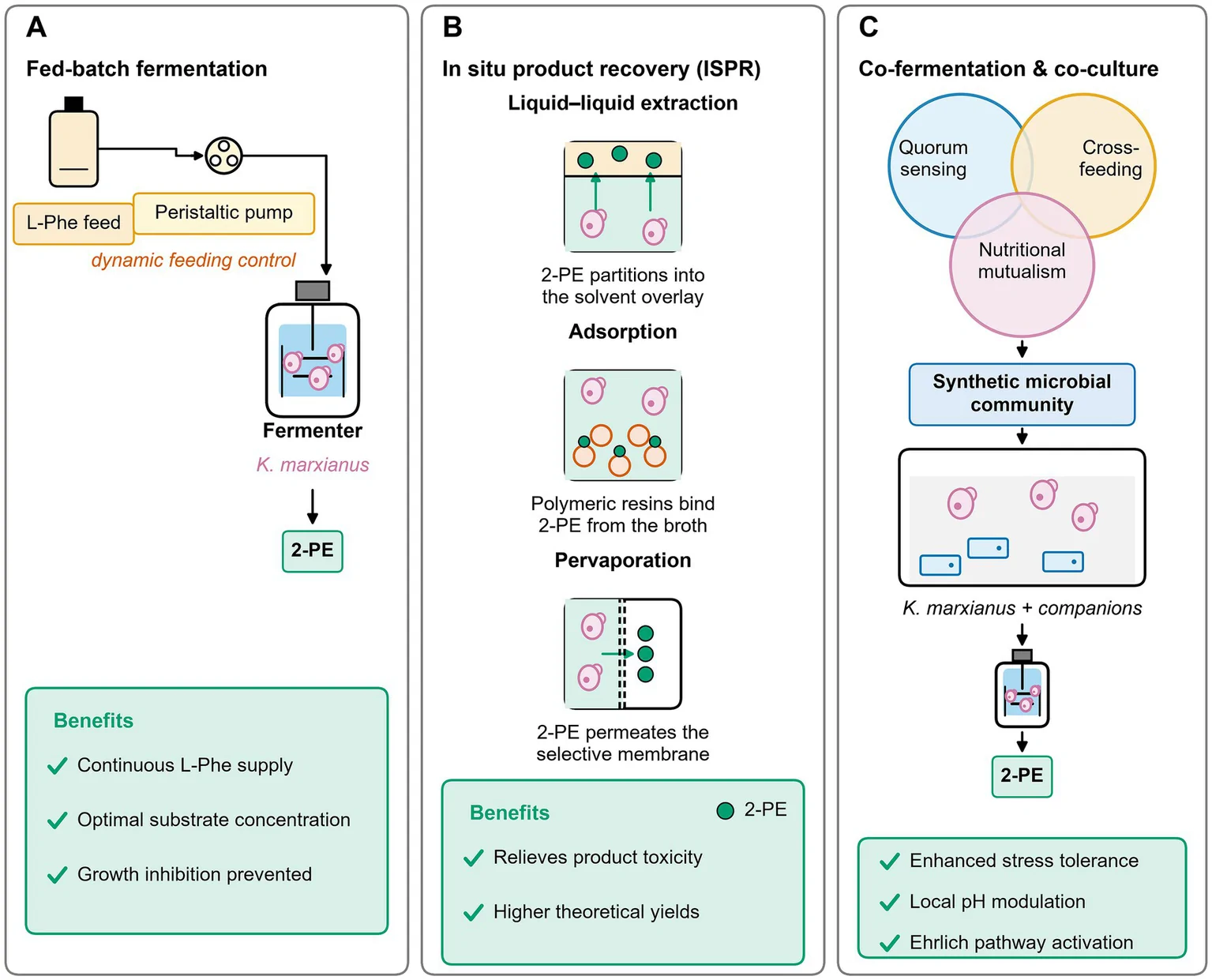
Different fermentation strategies for 2-PE production: **(A)** Fed-batch fermentation with dynamic feeding control for precursor (L-Phe) supply, maintaining optimal substrate concentration while preventing growth inhibition; **(B)** *In situ* product recovery (ISPR) using liquid–liquid extraction, adsorption, or pervaporation to continuously remove 2-PE from the fermentation broth, relieving product toxicity and enabling higher theoretical yields; **(C)** Co-fermentation and co-culture systems leveraging interspecies mutualism between *K. marxianus* and companion microorganisms to enhance stress tolerance, modulate local pH, and activate Ehrlich pathway gene expression.

Fed-batch fermentation with dynamic regulation is the most prevalent method in industrial applications (Figure 4A). In 2-PE fermentation, the primary challenge is developing intelligent feeding strategies for the precursor L-Phe. Excessive L-Phe inhibits cell growth, while insufficient amounts limit 2-PE production, making precise control of feeding rate and timing essential (Bernardino et al., 2024b). Feedback regulation, using online or offline monitoring of parameters like specific growth rate, dissolved oxygen levels, or residual substrate concentration, has been successfully applied. Recent studies have integrated dynamic regulation with metabolic engineering by creating gene expression systems responsive to L-Phe concentration or specific growth phases (Wang et al., 2017; Dai et al., 2021). These systems facilitate automatic transitions between “growth” and “production” stages, optimizing 2-PE synthesis while sustaining adequate biomass.

*In situ* product recovery (ISPR) is a crucial strategy for reducing 2-PE toxicity (Figure 4B). By removing the product from the medium during fermentation, ISPR alleviates cell inhibition, allowing the process to reach higher theoretical yields. The main ISPR methods for 2-PE include: (1) *In situ* liquid–liquid extraction involves adding organic solvents with high distribution coefficients for 2-PE and low microbial toxicity, such as oleic acid and decyl alcohol, to create a second phase that selectively extracts 2-PE (Brewster et al., 2025). The thermotolerance of *K. marxianus* enables extraction at elevated temperatures, enhancing mass transfer and product solubility. (2) *In situ* adsorption involves adding polymeric resins, especially hydrophobic adsorbents, directly into the fermenter or placing them in an external column to selectively bind 2-PE (Simko et al., 2015; Cervenansky et al., 2019). This method is straightforward to operate, allows for easy resin regeneration, and significantly boosts the overall 2-PE yield. (3) Pervaporation is a membrane-based technique that employs selective permeation under vacuum or carrier-gas conditions to preferentially remove and condense volatile 2-PE (Jankovic et al., 2025; Wang Y. H. et al., 2025). This method is both highly efficient and environmentally friendly. It complements the high-temperature fermentation characteristics of *K. marxianus*, significantly reducing energy consumption in downstream separation.

Co-fermentation and co-culture systems, inspired by natural ecological interactions, present an innovative approach (Figure 4C). Cultivating *K. marxianus* alongside one or more microorganisms enhances microenvironment efficiency through interspecies mutualism (Lukito et al., 2023). A notable example is the co-cultivation with lactic acid bacteria, such as *Pediococcus lactis*. Using this system, Han et al. (2024) demonstrated that both intact cells and isolated cell-wall fractions of *P. lactis* act as environmental signals that significantly activate key Ehrlich-pathway genes of *K. marxianus*, indicating that cell-wall-derived elicitors (e.g., peptidoglycan fragments, teichoic acids, or exopolysaccharides), rather than a soluble metabolite alone, mediate the stimulation. Plausible downstream mechanisms include: (i) elicitor-triggered stress responses that upregulate amino-acid catabolic genes (ARO9/ARO10 and their transcriptional activators, e.g., Aro80p homologs); (ii) local acidification by lactic acid, since mild acid stress is known to enhance Ehrlich-pathway flux; (iii) precursor cross-feeding through the release of free amino acids, including L-phenylalanine, via bacterial proteolysis and autolysis; and (iv) consumption or detoxification of inhibitory metabolites by the partner strain. The relative contribution of each mechanism remains to be quantified. These synthetic microecological systems can boost 2-PE production severalfold without complex genetic modifications. Similarly, co-culturing *K. marxianus* with *Debaryomyces hansenii* in whey-based medium doubled the 2-PE titer relative to single cultures, reaching 2.55 g/L (Castillo et al., 2022), an effect attributable to complementary substrate utilization and altered microenvironment rather than to direct genetic interaction. In a subsequent 2-L bioreactor scale-up using cheese whey supplemented with brewer’s spent yeast as a renewable nitrogen source, the same co-culture achieved a 2-PE productivity of 0.04 g/L/h while reducing the whey chemical oxygen demand below detection limits, thereby coupling aroma production with wastewater valorization (Castillo et al., 2023).

### 4.2 Utilization of low-cost substrates and culture media

Raw material costs significantly influence the economics of biomanufacturing. Utilizing affordable, renewable, and non-food biomass is crucial for process innovation, targeting both sustainability and economic viability.

Economic analyses and reviews of biotechnological 2-PE production indicate that raw-material expenses typically constitute an estimated 40–60% of total production costs, with downstream separation and purification contributing a further 20–30% (Qian et al., 2019; Bernardino et al., 2024b; Jankovic et al., 2025). Although microbial fermentation currently incurs higher estimated production costs (on the order of US$15–25/kg) than chemical synthesis (US$5–10/kg), natural certification commands a price premium of more than hundredfold. In *K. marxianus*-based processes, substituting glucose with low-cost feedstocks such as whey (4–5% lactose, negligible cost as a waste stream) or lignocellulosic hydrolysates is estimated to reduce medium-related costs by 60–80%. Thermotolerant fermentation at 40–45 °C additionally reduces cooling-energy demand—estimated at 30–40% relative to mesophilic operation at 30 °C, consistent with process-energy discussions for thermotolerant yeasts (Fonseca et al., 2008)—and pairs naturally with high-temperature ISPR. For economic competitiveness, indicative targets are titers above 10 g/L with integrated ISPR and productivity above 0.5 g/L/h on waste-derived feedstocks. We emphasize that these figures are indicative estimates synthesized from published analyses of comparable processes; a dedicated techno-economic analysis of a *K. marxianus* 2-PE plant has not yet been reported and represents an important future task.

Among the available options, utilizing whey efficiently highlights one of *K. marxianus*’s key strengths (Gao et al., 2024). Whey, a major by-product of cheese production, contains 4–5% lactose, proteins, and minerals. Its direct discharge poses significant environmental challenges. *K. marxianus* excels in lactose hydrolysis and metabolism via its intracellular β-galactosidase, making it ideal for whey valorization (Drezek et al., 2021). Recent research focuses on developing cost-effective pretreatment methods and optimizing fermentation in whey-based media to enhance 2-PE yields while reducing whey-related pollution (Alonso-Vargas et al., 2022).

Utilizing hydrolyzed lignocellulosic liquors marks a significant advancement. Sugars released from pretreated agricultural and forestry residues primarily include glucose and xylose, along with inhibitors like furfural and phenolic compounds. *K. marxianus* exhibits strong tolerance to these inhibitors and can metabolize glucose and xylose simultaneously, making it a promising candidate for lignocellulosic biorefining (Gao et al., 2017). Current research focuses on enhancing its tolerance to complex inhibitor mixtures through adaptive evolution or metabolic engineering. Efforts also aim to improve its coordinated use of pentoses and hexoses to ensure stable and efficient conversion of lignocellulose-derived sugars into 2-PE (Baptista et al., 2021).

### 4.3 Process integration and enhancement

Significant industrial breakthroughs typically arise from systematically integrating and optimizing multiple unit operations. This approach creates synergistic effects, making the whole greater than the sum of its parts.

*Kluyveromyces marxianus*’s ability to ferment at high temperatures is a significant advantage over other yeasts. Operating at 40–45 °C, or even higher, provides several benefits (Abdel-Banat et al., 2021; Ebe et al., 2025): (1) It reduces cooling needs, significantly cutting heat-exchange energy consumption in large fermenters; (2) It lowers contamination risks, as higher temperatures suppress most common contaminants, reducing the need for intense sterilization and antibiotics; (3) It accelerates reaction kinetics and mass transfer, enhancing biochemical conversions and product distribution during extraction; (4) It improves integration with downstream operations, allowing direct coupling with high-temperature separation processes like pervaporation, eliminating the need for intermediate cooling.

Integrating fermentation with separation exemplifies advanced process intensification. For instance, combining high-temperature fermentation with in-situ pervaporation enables *K. marxianus* to grow and produce 2-PE in a single high-temperature reactor (Etschmann et al., 2005). The volatilized 2-PE permeates a selective membrane, condenses, and is collected on the permeate side. This setup maintains a forward-driven reaction by continuously removing the product, theoretically allowing for continuous, long-term production and simplifying downstream purification. However, industrial implementation faces challenges such as ensuring long-term membrane biocompatibility, preventing fouling, and managing complex process control (Bernardino et al., 2024b).

## 5 Challenges and future perspectives

Despite significant advancements in engineering *K. marxianus* for 2-PE production, achieving economically viable industrial-scale yields remains challenging due to interconnected issues ranging from molecular mechanisms to process engineering. Identifying these bottlenecks and developing innovative solutions are crucial for the field’s continued progress (Figure 5).

**Figure 5:**
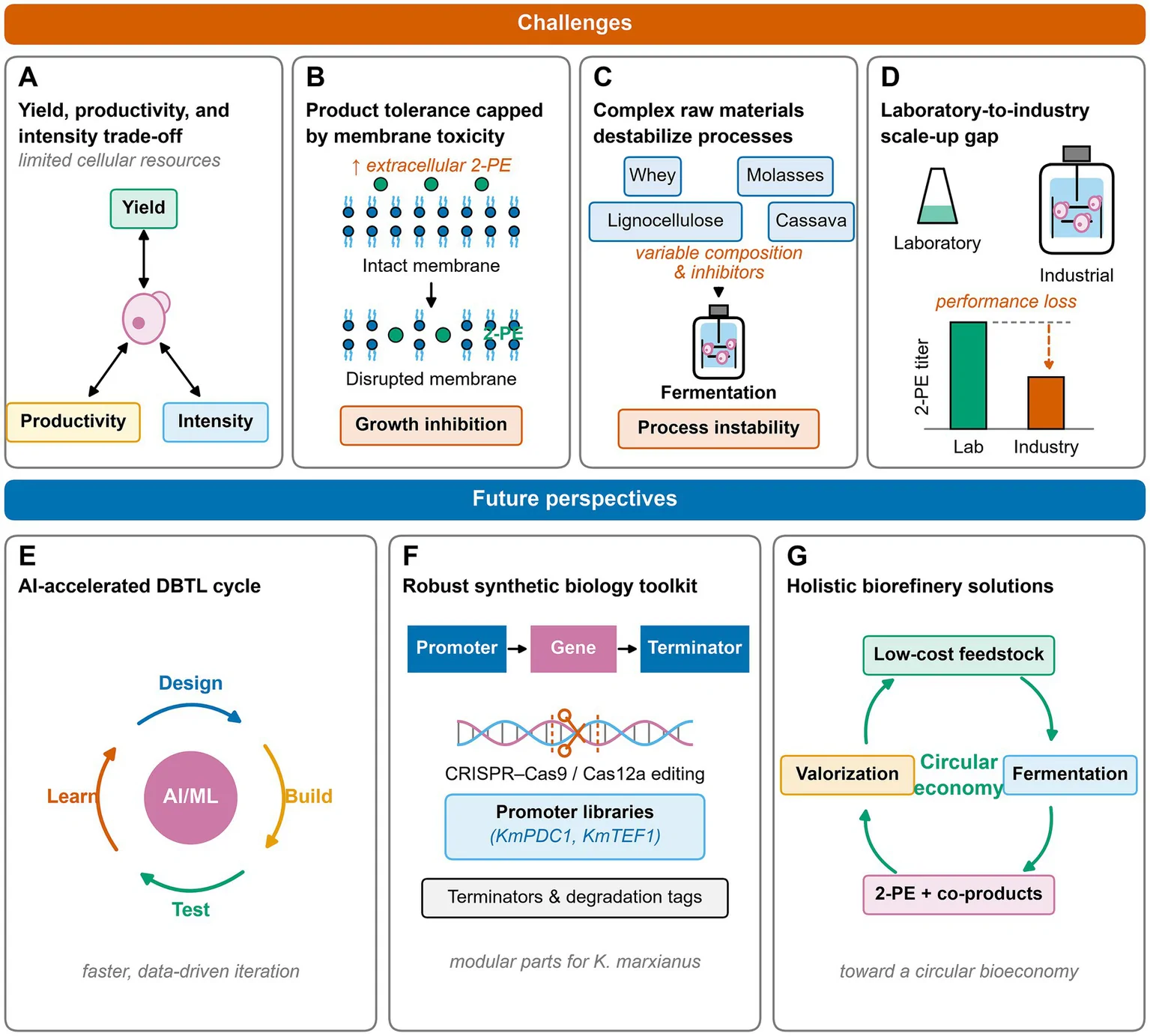
Challenges and future perspectives with 2-PE fermentation. **(A)** Trade-off between yield, productivity, and production intensity due to limited cellular resources. **(B)** Product tolerance limited by membrane toxicity. **(C)** Complexity of low-cost raw materials causing process instability. **(D)** Scale-up gap between laboratory and industrial performance. **(E)** AI-driven DBTL cycle acceleration. **(F)** Development of robust synthetic biology toolkit for *K. marxianus*. **(G)** Holistic biorefinery solutions for circular economy.

### 5.1 Current major bottlenecks

The trade-off between yield, productivity, and production intensity (Figure 5A) highlights the inherent conflict between cellular growth and product synthesis, a central challenge in metabolic engineering. A strain’s metabolic resources, such as carbon flux, energy, and reducing power, are limited. Allocating significant portions of these resources to 2-PE biosynthesis compromises growth and viability, extends fermentation time, and decreases biomass accumulation. While two-stage “growth–production” strategies offer some relief, achieving an optimal balance between high productivity and production intensity necessitates more advanced dynamic regulatory systems. Furthermore, the theoretical yield of converting exogenous L-Phe via the Ehrlich pathway is inherently limited. Constructing a complete *de novo* pathway from sugars to 2-PE is also challenging due to its length and low energetic efficiency.

Quantitative analysis of reported strains shows significant trade-offs among titer, productivity, and yield; however, because the underlying studies differ considerably in host, substrate (glucose-only versus L-phenylalanine-supplemented), fermentation mode, and use of ISPR, these values should be viewed as indicative benchmarks rather than a strictly comparable ranking. For instance, engineered *S. cerevisiae* JM2014 reached a titer of 9.79 g/L with a productivity of 135.97 mg/L/h using biphasic extraction (Chreptowicz and Mierzejewska, 2018). In contrast, *K. marxianus* KmTY4 produced 1.30 g/L at 18 mg/L/h. The highest reported 2-PE titer, 14.0 g/L, was achieved by *Metschnikowia pulcherrima* through continuous fermentation with solid-phase extraction, but required extended fermentation time (Chantasuban et al., 2018). These findings indicate that high titers generally necessitate ISPR integration, and that *K. marxianus* currently trails other hosts in both titer and productivity, highlighting the need for intensified engineering efforts. The theoretical maximum yield of *de novo* synthesis from glucose, calculated from pathway stoichiometry—two phosphoenolpyruvate plus one erythrose-4-phosphate (10 carbons) yield one 2-PE molecule (8 carbons) with two carbons lost as CO_2_—is approximately 0.26 g 2-PE/g glucose (≈0.38 mol/mol, i.e., ≈51% of theoretical carbon yield), consistent with values reported for *de novo* production of shikimate-pathway derivatives. Reported *de novo* titers and yields, such as those above, remain well below this theoretical maximum, indicating substantial room for pathway optimization.

The upper limit of production is constrained by the physicochemical toxicity of 2-PE toward the cell membrane (Figure 5B). The hydrophobic nature of 2-PE disrupts membrane integrity. Although lipid-composition engineering and efflux-pump engineering can enhance tolerance, these approaches face fundamental biophysical limitations. In planktonic cultures without product removal, extracellular 2-PE concentrations above approximately 4–8 g/L already strongly inhibit the growth and metabolism of most production hosts, including *S. cerevisiae* and *K. marxianus* (Balbino et al., 2021; Holyavkin et al., 2023; Brewster et al., 2025). The highest reported titers—approximately 10–15 g/L (up to ~17 g/kg in continuous extractive culture)—were achieved only in processes that integrated ISPR [e.g., 9.79 g/L with biphasic extraction (Chreptowicz and Mierzejewska, 2018); 14.0 g/L with continuous solid-phase extraction (Chantasuban et al., 2018); 16.6 g/kg with continuous extractive fermentation (Brewster et al., 2025)], which maintains the residual aqueous concentration below inhibitory levels. The 10–15 g/L range therefore reflects values attained in specific ISPR-assisted microbial systems rather than a general tolerance limit, and it may shift upward as tolerance engineering and process intensification advance. Improving tolerance alone may impose adaptive burdens that reduce cell fitness; addressing this tolerance–viability trade-off remains a major challenge.

The complexity of low-cost raw materials poses challenges to process robustness (Figure 5C). Economical feedstocks like whey and lignocellulosic hydrolysates are crucial for cost-effective production. However, their compositional complexity and batch variability undermine process stability and reproducibility. Inhibitors in lignocellulosic hydrolysates, such as furan aldehydes, phenolics, and organic acids, can synergistically increase toxicity, even affecting the relatively tolerant *K. marxianus*. This results in delays and fluctuations in fermentation performance. Therefore, developing industrial strains with broad adaptability to diverse crude feedstocks and flexible process conditions is vital for successful scale-up.

The transition from laboratory performance to industrial scale-up presents significant challenges (Figure 5D). Strategies that succeed in shake flasks or small fermenters often falter at larger scales. *In situ* product removal methods, such as extraction and adsorption, encounter obstacles in industrial settings, including decreased phase-separation efficiency, higher material costs, increased operational complexity, and sterility issues. Additionally, maintaining stable population dynamics in co-fermentation systems becomes more challenging as the scale increases. Variations in mixing, mass transfer, and heat transfer at industrial volumes create heterogeneous microenvironments, potentially compromising overall productivity.

### 5.2 Future research directions

Future research will increasingly adopt systematic, intelligent, and sustainable strategies to address these challenges. This shift will advance the field from linear optimization to multidimensional, integrated design.

Artificial intelligence and automation are accelerating the design-build-test-learn (DBTL) cycle (Peterman et al., 2024) (Figure 5E). Integrating machine learning with automated laboratories significantly speeds up strain engineering (Ghosh et al., 2024). AI models that combine multi-omics data, literature, and phenotype datasets can predict high-performance genotypes and suggest effective editing strategies (Ding et al., 2025). Automated robotic systems, like liquid-handling platforms and high-throughput microreactors, facilitate rapid experimental validation (Chory et al., 2021; Zhang et al., 2023). The data generated are then used to refine models iteratively, creating an efficient, data-driven DBTL loop. This process enhances the success of rational design and broadens the accessible genetic landscape (Pretorius et al., 2025).

Developing a robust and universal synthetic biology toolkit for *K. marxianus* is essential (Figure 5F). Compared to *S. cerevisiae* and *Escherichia coli* (Xiong et al., 2025), tools for *K. marxianus* are still limited. Future efforts should concentrate on creating more efficient CRISPR systems compatible with various wild-type strains. Additionally, there is a need for comprehensive libraries of standardized biological parts, such as promoters, terminators, and degradation tags, along with tools for controlling protein localization and interactions. A modular, predictable, plug-and-play toolkit would lower engineering barriers and facilitate the construction of complex genetic circuits.

In our assessment, the feasibility of these directions varies with their technical maturity. AI-driven DBTL cycles appear achievable in the near term, given rapid advances in automated laboratory platforms and machine-learning algorithms, although their application to *K. marxianus* will require substantial accumulation of training data. A comprehensive synthetic-biology toolkit for *K. marxianus* is achievable in the medium term but will require a coordinated community effort, as evidenced by a decade of tool development for *S. cerevisiae*. Holistic biorefinery integration is a long-term goal that poses the greatest challenge, demanding simultaneous optimization of multiple product pathways, process integration, and economic viability. A bottleneck common to all directions is the limited availability of high-quality, standardized multi-omics data for *K. marxianus* under industrially relevant conditions, underscoring the urgent need for systematic data-generation efforts. These maturity assessments reflect the authors’ perspective rather than a formal technology-readiness evaluation.

Developing holistic biorefinery solutions focused on the circular economy and sustainability is crucial (Figure 5G). Research should transcend single-product approaches to maximize resource value and minimize environmental impact. For *K. marxianus*, a promising strategy involves multi-product co-production within integrated biorefineries. For instance, during whey processing, engineered strains could simultaneously produce 2-PE, single-cell protein, and lactase, fully valorizing all components (Yadav et al., 2015; Amini et al., 2025; Günal-Köroglu et al., 2025). Similarly, in lignocellulosic conversion, glucose can be directed toward 2-PE production, while xylose is used for ethanol or organic acids (Ren et al., 2024; Fortuin et al., 2025). These “one strain, multiple products” or “one feedstock, multiple outputs” strategies significantly enhance economic resilience and sustainability.

## 6 Conclusion

*Kluyveromyces marxianus* has become a promising microbial chassis for natural 2-PE biomanufacturing due to its thermotolerance, rapid growth, substrate versatility, and ability to catabolize aromatic amino acids. This review outlines a detailed roadmap to harness its biotechnological potential through integrated engineering strategies. At the intracellular level, metabolic engineering and systems biology enable precise flux redistribution, pathway optimization, and improved cellular robustness. At the bioprocess level, innovative fermentation strategies, such as advanced reactor operations, *in situ* product recovery, and sustainable low-cost feedstock use, address technical challenges, reduce costs, and promote circular bioeconomy principles.

Despite significant technological advances, achieving economically viable and industrially competitive 2-PE production still requires addressing persistent challenges. These include maximizing titer, enhancing product tolerance, ensuring feedstock robustness, and developing scalable bioprocess engineering. Future progress relies on integrating synthetic biology, systems biology, and process engineering. This integration is crucial for developing dynamic, self-regulating metabolic circuits, AI-driven predictive strain design platforms, and holistic biorefinery concepts that align with circular economy and sustainability frameworks. By adopting comprehensive, end-to-end integrated design paradigms—from molecular-level pathway engineering to factory-scale bioprocess implementation—engineered *K. marxianus* can become a highly efficient microbial cell factory for 2-PE production. This approach also sets a precedent for innovative, sustainable practices in next-generation industrial biotechnology.

Emerging technologies are poised to advance *K. marxianus*-based 2-PE production. The CRISPR-Cas12a system, recently adapted for *K. marxianus*, enables multiplexed genome editing with greater flexibility than Cas9 and is expected to accelerate the construction of complex metabolic pathways (Zha et al., 2026). Adaptive laboratory evolution combined with biosensor-assisted selection has recently been shown to resolve tolerance–efficiency trade-offs in other production hosts (Zhang Y. F. et al., 2025) and represents a promising—but not yet demonstrated—route to higher 2-PE tolerance in *K. marxianus*. Dynamic metabolic control circuits that respond to product concentration or growth phase have been demonstrated in other microorganisms and could, upon transfer to *K. marxianus*, help balance the growth–production trade-off with less manual intervention. Likewise, *K. marxianus*-specific genome-scale metabolic models are expected to facilitate in silico design of engineering strategies and reduce empirical screening, although their 2-PE-specific application remains to be demonstrated. Addressing key scientific questions—such as leveraging thermotolerance mechanisms for product tolerance and fine-tuning carbon catabolite repression for optimal substrate co-utilization—will be crucial for realizing the full industrial potential of *K. marxianus* in 2-PE biomanufacturing.
